# Beyond bacteria: a multi-omics view of the gut–brain axis in Parkinson’s disease

**DOI:** 10.3389/fcimb.2026.1900578

**Published:** 2026-09-11

**Authors:** Huiye Han, Owen Luo, Kevin Li, Shyazana Rahaman, Eunice Unbyul Ok, Liangliang Zhang, Menglu Liang, Huang Lin

**Affiliations:** 1 Department of Mathematics, University of Maryland, College Park, MD, United States; 2 Winston Churchill High School, Potomac, MD, United States; 3 Department of Epidemiology and Biostatistics, University of Maryland, College Park, MD, United States; 4 Department of Population and Quantitative Health Sciences, School of Medicine, Case Western Reserve University, Cleveland, OH, United States

**Keywords:** gut–brain axis, metabolomics, microbiome, multi-omics, mycobiome, Parkinson’s disease, proteomics, virome

## Abstract

**Introduction:**

Parkinson's disease (PD) is increasingly recognized as a multisystem disorder in which gastrointestinal dysfunction and gut microbial alterations may contribute to disease pathophysiology. Although most microbiome research in PD has focused on bacteria, growing evidence suggests that the gut ecosystem should be considered more broadly to include fungi, viruses, metabolites, and proteins.

**Methods:**

We searched PubMed and SciFinder for human studies published up to October 28, 2025, using domain-specific search strategies for the bacteriome, metabolome, proteome, virome, and mycobiome, and synthesized the eligible evidence using a structured multi-omics evidence-mapping framework.

**Results:**

We summarize the most consistent bacterial findings, including enrichment of mucin-degrading taxa and depletion of short-chain fatty acid-producing commensals, and discuss how these changes relate to impaired fermentation, barrier dysfunction, and immune activation. We further examine emerging evidence for virome and mycobiome alterations, highlighting the possibility that PD-related dysbiosis reflects cross-kingdom ecological disruption rather than bacteria-only imbalance. Metabolomic studies provide functional support for this model by demonstrating altered short-chain fatty acid biology and broader host -microbe co-metabolic remodeling. Protein-focused studies, including host proteomic signatures and bacterial functional amyloids, extend the field toward mechanisms linking gut dysfunction to inflammation, proteostatic stress, and α-synuclein pathology.

**Discussion:**

Overall, the evidence supports a multi-layer view of the PD gut -brain axis in which microbial ecology, metabolic output, barrier integrity, immune signaling, and protein-centered mechanisms are interconnected. The field remains limited by cross-sectional designs, methodological heterogeneity, and uneven evidence depth across omics layers. Longitudinal, standardized, and integrated multi-omics studies will be essential to determine which microbiome-associated alterations are mechanistically important, clinically informative, and potentially modifiable in PD.

## 1 Introduction

Parkinson’s disease (PD) is the second most common neurodegenerative disorder and an increasing global public health challenge. A 2025 Global Burden of Disease modeling study projected that the number of people living with PD will reach 25.2 million worldwide by 2050, underscoring the need to better understand disease mechanisms and identify modifiable pathways (Su et al., 2025). Although PD is classically defined by bradykinesia, rigidity, tremor, and postural instability, it is now recognized as a multisystem disorder with prominent non-motor features, including constipation and other gastrointestinal symptoms that may precede motor diagnosis by years (Braak et al., 2003). This temporal sequence has helped motivate the gut-first hypothesis of PD, in which peripheral events in the gastrointestinal tract, enteric nervous system, mucosal immune compartment, and their microbial milieu may contribute to downstream neuroinflammation, *α*-synuclein misfolding, and neurodegeneration (Braak et al., 2003; Bedarf et al., 2017; Wang et al., 2021).

Over the past decade, studies of the PD gut microbiome have focused predominantly on bacteria, and several meta-analyses now support the presence of reproducible but modest disease-associated shifts in microbial composition. Across cohorts, enrichment of genera such as *Akkermansia*, *Lactobacillus*, and *Bifidobacterium*, together with depletion of short-chain fatty acid (SCFA)-producing taxa including members of Lachnospiraceae and *Faecalibacterium*, has emerged as one of the most consistent patterns (Nishiwaki et al., 2020; Romano et al., 2021; Boktor et al., 2023). Multi-omics studies further suggest that the PD-associated gut ecosystem is characterized not only by taxonomic dysbiosis but also by altered microbial function, metabolite production, intestinal inflammation, and host responses (Tan et al., 2021; Villette et al., 2025). However, bacterial findings remain heterogeneous across studies, likely reflecting differences in geography, diet, medication exposure, constipation, sequencing strategy, bioinformatic pipelines, and study design (Nishiwaki et al., 2020; Romano et al., 2021; Boktor et al., 2023).

At the same time, a bacteria-only framework is no longer sufficient to explain gut–brain signaling in PD. The intestinal ecosystem also includes fungi, viruses, and a broad array of microbial- and host-derived metabolites and proteins that shape mucosal homeostasis, barrier integrity, immune tone, and proteostasis. Recent PD studies have therefore started to examine the gut virome (Bedarf et al., 2017; Chen et al., 2026), the fecal mycobiome alongside metabolic change (De Pablo-Fernandez et al., 2022), and metabolomic and proteomic signatures that may connect gut dysbiosis with systemic inflammation, neurotransmitter-related pathways, and prodromal biomarker development (Augustin et al., 2023; Hällqvist et al., 2024; Villette et al., 2025). Meanwhile, work on bacterial functional amyloids raises the possibility that microbial proteins may directly contribute to cross-seeding or amplification of *α*-synuclein pathology, extending microbiome research in PD beyond descriptive community profiles alone (Wang et al., 2021; Wojciechowska et al., 2024).

Taken together, these developments raise a central question: how can findings across bacteria, fungi, viruses, metabolites, and proteins be assembled into a coherent model of PD biology? Several gaps still hinder that effort. First, the evidence base is uneven, with bacterial 16S studies far outnumbering virome, mycobiome, and proteome investigations. Second, most studies remain cross-sectional and observational, which limits causal inference. Third, omics layers are often studied separately even though the underlying biology is inherently cross-kingdom and host–microbe interactive. Finally, methodological heterogeneity and incomplete control of key confounders make it difficult to separate robust disease signals from cohort-specific effects (Nishiwaki et al., 2020; Romano et al., 2021; Tan et al., 2021; Villette et al., 2025).

Against this backdrop, this review synthesizes current evidence on the microbiome in PD through a broader multi-omics lens that includes bacteria, fungi, viruses, metabolites, and proteins. We focus on three questions: which alterations are most consistently associated with PD across these domains; what mechanistic links connect these signals to gut barrier dysfunction, inflammation, and neurodegenerative processes; and which evidentiary and methodological gaps must be addressed before the field can move toward clinically useful biomarkers and microbiome-informed interventions? By placing established bacterial findings alongside newer data on the mycobiome, virome, metabolome, and proteome, we aim to clarify both what is relatively secure and what remains unsettled in the expanding microbiome landscape of PD.

Throughout this review, we use the term microbiome in a broad sense to refer to the intestinal microbial ecosystem and its functional activity, rather than to bacteria alone. Where relevant, we use bacteriome to describe the bacterial component, virome for intestinal viruses, and mycobiome for the fungal component. We use metabolome and proteome to refer to metabolite- and protein-level molecular readouts that reflect host–microbe interactions and extend interpretation beyond taxonomic composition alone.

## 2 Materials and methods

### 2.1 Data sources and search strategy

This systematic review was conducted and reported in accordance with the PRISMA 2020 Statement (Page et al., 2021). We searched PubMed and SciFinder for studies published up to October 29, 2025. To capture the multi-omics landscape of PD, we developed separate free-text strategies for the bacteriome, metabolome, proteome, virome, and mycobiome. Search terms were tailored to each domain to reflect differences in terminology and research focus. The complete search formulas used for each omics domain are provided in **Supplementary Table 1**.

### 2.2 Study selection and eligibility criteria

Study selection followed a two-stage process. We first applied technical filters to retain human studies published in English with full-text availability and to exclude non-original publications, including reviews, case reports, dissertations, and conference abstracts. We then performed scientific screening through title and abstract review followed by full-text assessment. Screening was conducted by one reviewer and independently cross-checked by a second reviewer, with disagreements resolved through discussion.

Eligible studies were required to be original human research focused on PD and to report at least one relevant omics modality, including bacteriome, metabolome, proteome, virome, or mycobiome data. Because the evidence base differed substantially across omics domains, a tiered inclusion strategy was used.

#### 2.2.1 Inclusion and exclusion criteria for bacteriome and metabolome studies

For bacteriome and metabolome studies, where the literature is relatively mature, we applied stricter criteria. Observational studies in these domains were required to include a healthy control group, rather than only disease controls, enroll at least 50 participants per group, and report original biological findings rather than primarily predictive modeling or statistical methodology development. Microbiome studies were additionally required to report results at the genus or species level to ensure sufficient taxonomic resolution. Observational studies in these domains were assessed using the Newcastle–Ottawa Scale (NOS), and only studies with scores of 6 or higher were included. Interventional studies (e.g., randomized controlled trials and prebiotic, probiotic, or resistant starch supplementation studies) were not subject to the ≥50-per-group or NOS criteria, since those criteria are designed for case-control observational designs; instead, interventional studies were retained when they reported PD-relevant microbiome or metabolome outcomes in human participants.

The ≥50 participants-per-group threshold was applied to reduce small-sample bias and improve the stability and reproducibility of findings in bacteriome and metabolome studies, which are typically high-dimensional and exhibit substantial inter-individual variability. Although no universal cutoff exists, restricting analyses to studies with moderate sample sizes is a common practice in omics research to mitigate overfitting and enhance the robustness of identified biological signals.

The NOS ≥6 cutoff was used to retain studies of at least moderate methodological quality. While not formally standardized, this threshold is widely used in systematic reviews to indicate acceptable quality with respect to selection, comparability, and outcome assessment, thereby helping to limit bias from lower-quality observational studies.

#### 2.2.2 Inclusion and exclusion criteria for virome, mycobiome, and proteome studies

By contrast, proteome, virome, and mycobiome studies remain comparatively underexplored, so we did not impose additional sample size or design restrictions beyond the general criteria. All studies meeting the general inclusion criteria were retained to avoid excluding emerging but potentially informative evidence. This approach was intended to balance methodological rigor in more established areas with inclusiveness in newer fields.

**Figure 1:**
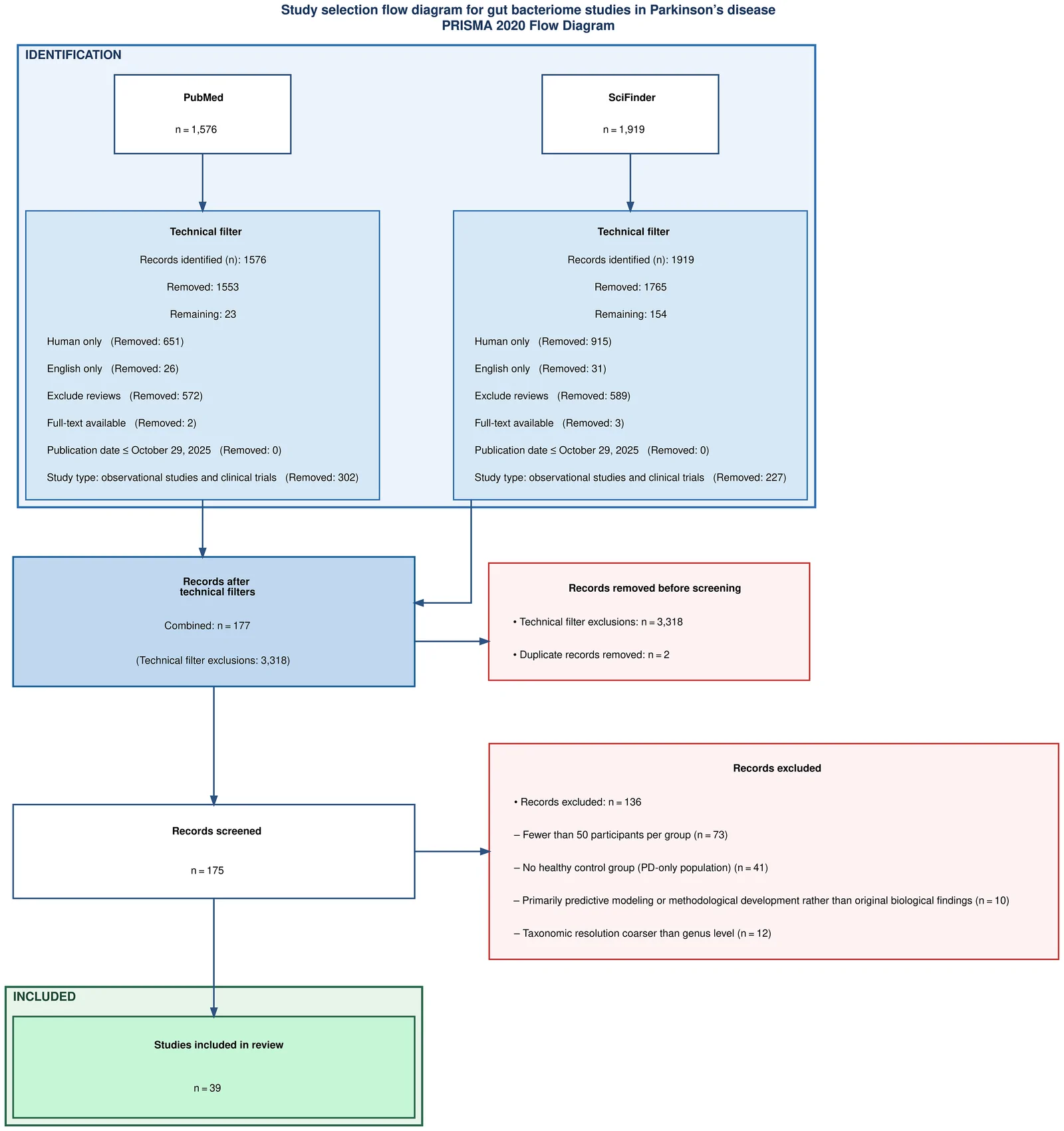
PRISMA flow diagram for gut bacteriome study identification and selection. The diagram summarizes database searching, technical filtering, duplicate removal, eligibility screening, and final study inclusion for gut microbiome studies.

Studies were excluded if they were not specific to PD, did not report PD-relevant outcomes, involved animal or *in vitro* experiments, or were non-original publications. We also screened the reference lists of included studies to identify additional eligible articles. The selection process for the microbiome (bacteriome) studies is summarized in the PRISMA flow diagram (**Figure 1**).

A formal review protocol was developed by the authors, but the review was not prospectively registered because the multi-omics scope and domain-specific inclusion criteria were refined iteratively during protocol development. To support transparency and reproducibility, the full list of included studies (**Supplementary Table 2**), the PRISMA screening workflows for each omics domain except the bacteriome (**Supplementary Figures 1–4**), and the distribution of included studies by publication year and omics category (**Supplementary Figure 5**) are provided in the **Supplementary Materials**, and the review was conducted and reported in accordance with the PRISMA 2020 Statement.

### 2.3 Data extraction and quality assessment

Data were extracted using a predefined template that captured study characteristics, including author, publication year, country, study design, sample size, participant characteristics, omics modality, and key findings. Extraction was performed by one reviewer and independently verified by a second reviewer, with discrepancies resolved by discussion.

Quality assessment was performed according to study design. Randomized controlled trials were evaluated using the Cochrane Risk of Bias Tool, whereas observational studies were assessed with the NOS. As noted above, only observational studies with NOS scores of 6 or higher were included where this threshold was applied. Potential sources of bias were also considered qualitatively during interpretation.

**Figure 2:**
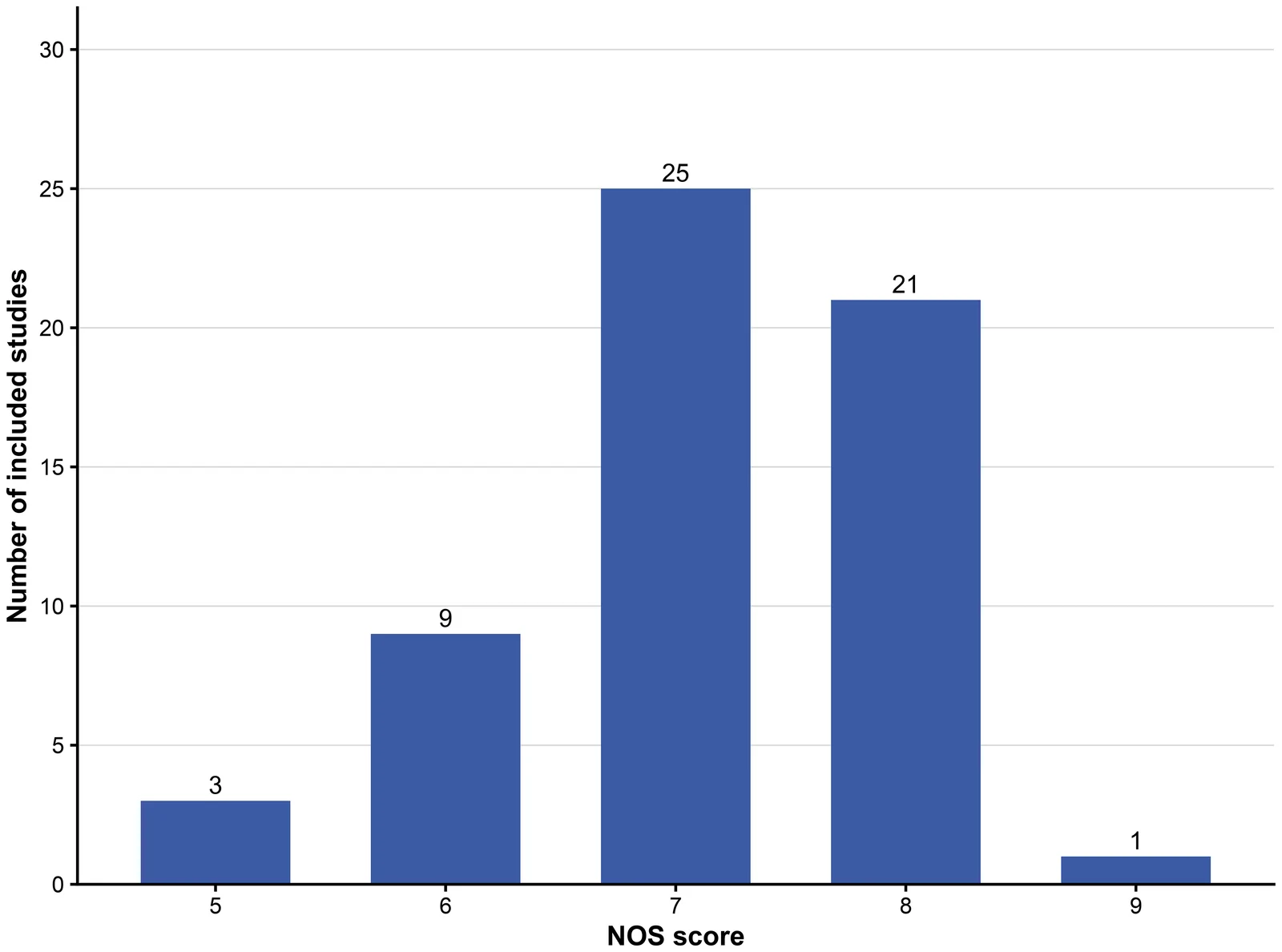
Distribution of Newcastle–Ottawa Scale (NOS) scores among included studies with available quality assessment. The bar chart summarizes the number of included studies at each NOS score. Most studies clustered in the moderate-to-high quality range, supporting the overall methodological rigor of the observational evidence base while also reflecting variation across study designs and omics domains.

The distribution of available NOS scores across included studies is shown in **Figure 2**. Most scored studies clustered in the moderate-to-high quality range, although quality-assessment procedures differed across omics domains and were not applicable to every included study design.

### 2.4 Evidence synthesis

Because of substantial heterogeneity in study populations, designs, and analytical methods, a quantitative meta-analysis was not performed. Instead, we used a structured evidence-mapping approach. Studies were first grouped by omics domain (bacteriome, metabolome, proteome, virome, and mycobiome) and by study type (observational or interventional). Findings were then organized into predefined biological themes, including microbial composition, metabolic alterations, and clinical associations. Consistencies and discrepancies across studies were evaluated with attention to reproducibility and likely sources of heterogeneity. This approach allowed integration of diverse multi-omics evidence while preserving domain-specific methodological context.

## 3 Results

### 3.1 Overview of included evidence

**Figure 3:**
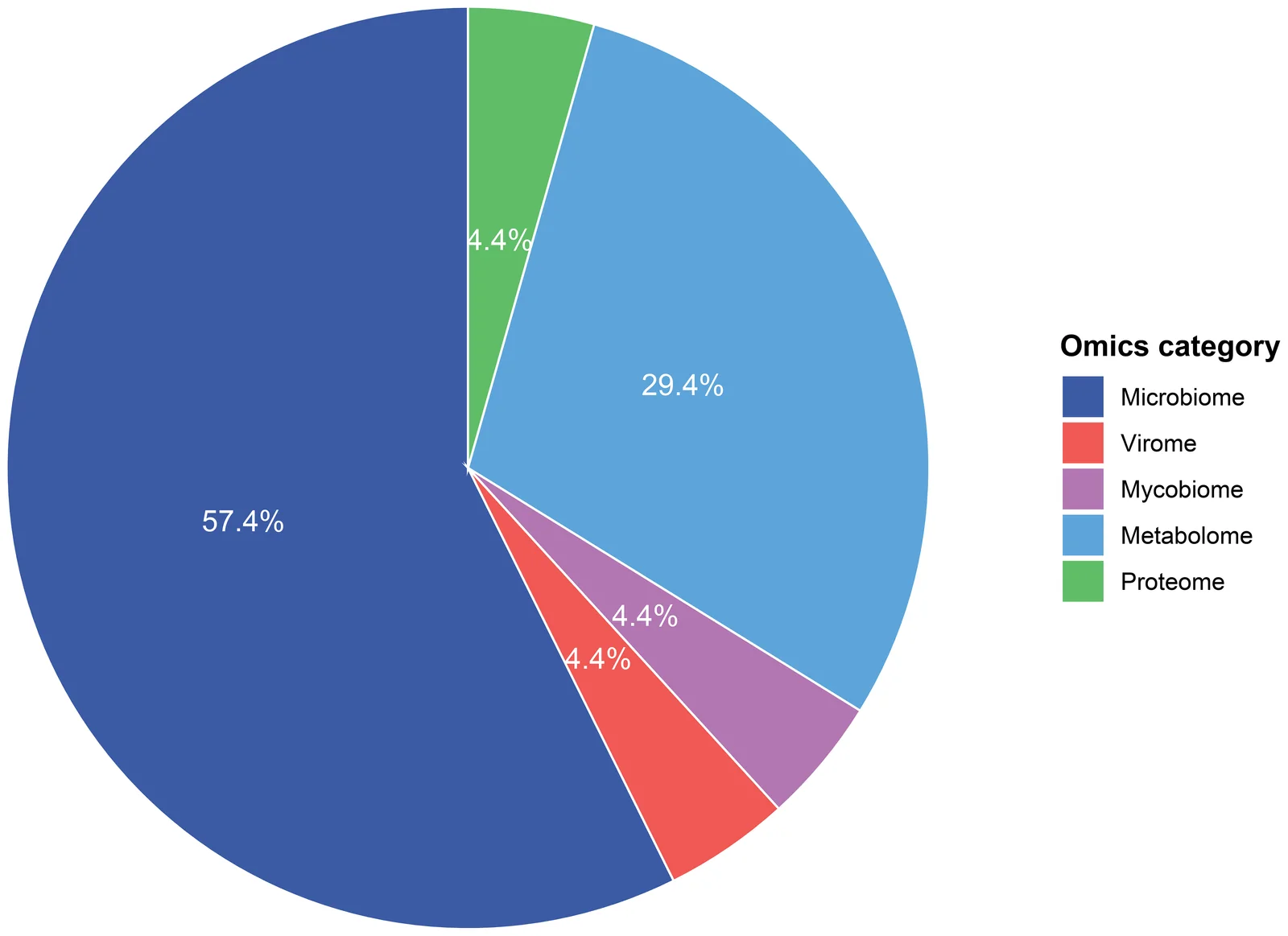
Distribution of included studies across omics domains in Parkinson’s disease. Pie chart showing the proportion of included studies classified as microbiome (bacteriome), virome, mycobiome, metabolome, and proteome. The figure highlights the marked imbalance in the current evidence base, with bacterial microbiome and metabolomic studies accounting for most included publications.

The included literature showed a markedly uneven evidence base across omics domains. Bacterial microbiome and metabolomic studies accounted for most of the available human data and included the largest case-control cohorts, the greatest number of replication studies, and most of the intervention literature (Nishiwaki et al., 2020; Romano et al., 2021; Tan et al., 2021; Boktor et al., 2023). In contrast, virome, mycobiome, and protein-focused studies were fewer, more heterogeneous in design, and often exploratory in scope (Bedarf et al., 2017; De Pablo-Fernandez et al., 2022; Hällqvist et al., 2024; Villette et al., 2025; Wojciechowska et al., 2024). The relatively small sample sizes of several studies in these emerging omics domains may reduce statistical power and increase sampling variability, leading to unstable effect estimates and cohort-specific findings. These limitations, together with differences in study design and analytical methods, may further contribute to between-study heterogeneity. Therefore, findings from these domains should be interpreted as exploratory and require validation in larger, independent cohorts. Across domains, most studies were observational and cross-sectional, with fewer longitudinal analyses and only a limited number of intervention trials (Aho et al., 2019; Becker et al., 2022; Hall et al., 2023; Petrov et al., 2025; Hegelmaier et al., 2026; Leta et al., 2025). In total, 68 study entries were included across omics categories, comprising 39 bacteriome, 3 virome, 3 mycobiome, 20 metabolome, and 3 proteome entries, quantitatively reflecting the uneven distribution of evidence across omics domains. Because 7 multi-omics studies report findings in more than one domain, these category counts include cross-listings and correspond to 61 unique studies (overlaps: 5 between bacteriome and metabolome, 1 between bacteriome and virome, and 1 between mycobiome and metabolome; see **Supplementary Table 2**). This imbalance is visualized in **Figure 3**, which highlights the predominance of bacteriome and metabolome studies in the current literature.

Accordingly, the evidence below is synthesized by omics layer, with emphasis on recurring observational findings, mechanistic implications, and the relative maturity of each field.

### 3.2 Gut bacteriome in PD

#### 3.2.1 Definition and scope

The gut bacterial microbiome is the most extensively studied microbial component in PD, largely because it can be profiled reproducibly using 16S rRNA sequencing, shotgun metagenomics, and integrated multi-omics approaches (Nishiwaki et al., 2020; Romano et al., 2021; Tan et al., 2021; Boktor et al., 2023). As a result, bacterial studies currently provide the strongest evidence for disease-associated gut dysbiosis and form the foundation for most mechanistic models of the PD gut–brain axis.

#### 3.2.2 Observational evidence: reproducible but modest compositional shifts

A large body of case-control, longitudinal, and meta-analytic work indicates that PD is associated with reproducible, but generally modest, alterations in gut bacterial composition, with small effect sizes and substantial overlap between PD and control groups (Hill-Burns et al., 2017; Lin et al., 2018, 2019; Aho et al., 2019; Heintz-Buschart et al., 2018; Chen et al., 2022a; Nishiwaki et al., 2020; Romano et al., 2021; Boktor et al., 2023). Across cohorts, recurrent findings include enrichment of *Akkermansia*, *Lactobacillus*, and *Bifidobacterium*, together with depletion of taxa linked to SCFA production, including members of Lachnospiraceae (e.g., *Roseburia* and *Blautia*) and *Faecalibacterium* (Hill-Burns et al., 2017; Lin et al., 2018, 2019; Mertsalmi et al., 2017; Aho et al., 2019; Heintz-Buschart et al., 2018; Nishiwaki et al., 2020; Romano et al., 2021). Shotgun metagenomic studies have sharpened this picture, with multiple reports describing enrichment of opportunistic pathogens such as *Escherichia coli* and *Klebsiella pneumoniae*, alongside depletion of SCFA-producing species such as *Roseburia intestinalis* and *Blautia wexlerae*. These patterns suggest that at least part of the PD-associated bacterial signature persists after accounting for confounders such as medication exposure and constipation (Wallen et al., 2022; Boktor et al., 2023; Duru et al., 2024; Palacios et al., 2023). Even so, between-study heterogeneity remains substantial, reflecting variation in geography, diet, stool consistency, analytical pipelines, and clinical characteristics (Nishiwaki et al., 2020; Romano et al., 2021; Boktor et al., 2023).

#### 3.2.3 Functional remodeling beyond taxonomy

Beyond taxonomic differences, recent studies suggest that PD-associated dysbiosis is better understood as functional remodeling of the gut ecosystem (Cirstea et al., 2020; Qian et al., 2020; Tan et al., 2021; Villette et al., 2025; Hensen and Thiele, 2025). Integrated metagenomic and metabolomic analyses indicate reduced carbohydrate fermentation capacity, increased proteolytic metabolism, and altered microbial pathways involving aromatic amino acids, bile acids, indole compounds, and levodopa metabolism (Cirstea et al., 2020; Qian et al., 2020; Cirstea et al., 2023; Tan et al., 2021; Hensen and Thiele, 2025). Metatranscriptomic evidence further suggests that functional and transcriptional shifts may be evident even when abundance changes are small, supporting the idea that microbial activity, rather than composition alone, is central to PD-associated gut dysfunction (Villette et al., 2025).

#### 3.2.4 Mechanistic pathways: barrier dysfunction, inflammation, and gut–brain signaling

Several bacterial findings converge on mechanistic models centered on intestinal barrier dysfunction and peripheral immune activation. Reduced abundance of butyrate-producing taxa, including *Faecalibacterium prausnitzii*, *Roseburia* spp., and members of the Lachnospiraceae and Ruminococcaceae families, is consistently linked to lower fecal SCFA concentrations, particularly butyrate, which may compromise epithelial integrity, alter mucosal immunity, and increase exposure to inflammatory microbial products (Aho et al., 2021; Chen et al., 2022a; Cirstea et al., 2020; Romano et al., 2021). At the same time, enrichment of mucin-degrading organisms such as *Akkermansia* may contribute to mucus layer thinning and greater epithelial contact with microbial-associated molecular patterns and lipopolysaccharide (Lin et al., 2019; Nishiwaki et al., 2020; Pietrucci et al., 2019; Chen et al., 2022b). Together, these changes support a model in which bacterial dysbiosis promotes barrier vulnerability, low-grade gut inflammation, and chronic immune signaling with potential consequences for neuroinflammatory pathways relevant to PD (Aho et al., 2021; Chen et al., 2022a; Villette et al., 2025).

#### 3.2.5 Interventional evidence

Interventional studies targeting the bacterial microbiome remain limited but provide preliminary evidence that the intestinal ecosystem in PD is biologically modifiable (Becker et al., 2022; Hall et al., 2023; Petrov et al., 2025; Hegelmaier et al., 2026; Leta et al., 2025; Du et al., 2025). Probiotics (e.g., *Lactobacillus* and *Bifidobacterium* strains), prebiotics (e.g., inulin, resistant maltodextrin, rice bran, and 2’-fucosyllactose), resistant starch, and SCFA-directed strategies (e.g., direct supplementation of propionic and butyric acid) have been associated with short-term improvements in constipation, inflammatory markers, microbial function, and in some studies motor or non-motor symptoms (Becker et al., 2022; Hall et al., 2023; Petrov et al., 2025; Hegelmaier et al., 2026; Leta et al., 2025; Du et al., 2025). However, most trials are small, heterogeneous, and of limited duration, so current evidence supports symptomatic and mechanistic plasticity more strongly than durable disease modification.

#### 3.2.6 Transitional summary

Overall, bacterial studies provide the most consistent evidence for gut ecosystem disruption in PD. Even so, the emerging picture is no longer limited to isolated taxa shifts. Instead, bacterial dysbiosis appears to intersect with altered metabolites such as SCFAs, ceramides, and glutamate, host immune responses including increased intestinal permeability and mucosal inflammation, and protein-level pathways involving microbial chemotaxis, flagellar assembly, and glutamate metabolism that become more apparent when multiple omics layers are considered together (Tan et al., 2021; Villette et al., 2025).

### 3.3 Gut virome in PD

#### 3.3.1 Definition and scope

The gut virome comprises bacteriophages and eukaryotic viruses that inhabit the intestinal ecosystem and influence bacterial ecology, horizontal gene transfer, host immunity, and mucosal homeostasis (Spencer et al., 2022). In PD, virome research remains at an early stage compared with bacteriome studies, but shotgun metagenomics has begun to reveal disease-associated viral features that may represent an additional layer of gut ecosystem disruption (Bedarf et al., 2017; Chen et al., 2026; Park et al., 2026).

#### 3.3.2 Observational evidence

Available virome studies suggest that PD is associated with altered viral community structure, although the findings remain preliminary and method-dependent. Early metagenomic work in L-DOPA-naïve PD reported reduced total viral signal relative to controls, but the analysis relied heavily on reference-based annotation and likely underestimated uncharacterized viral diversity (Bedarf et al., 2017). More recent studies using higher-resolution virome profiling report increased phage richness and evenness in PD, along with shifts in family-level composition, including decreases in Microviridae and Podoviridae and increases in Myoviridae, Siphoviridae, and Tectiviridae (Park et al., 2026). We note that the direction of change reported for Podoviridae in that study differs between the abstract, which lists the family among those enriched in PD, and the results text, which describes it as reduced; we follow the results text here (Park et al., 2026). Assembly-based analyses further identify substantial viral operational taxonomic unit (vOTU)-level differences. Hundreds of vOTUs are differentially abundant between PD and controls, most of them enriched in PD and frequently assigned to the Siphoviridae and Myoviridae families, supporting disease-related restructuring rather than random variability (Chen et al., 2026, 2022b). Even so, the small number of cohorts and differences in viral detection pipelines make it difficult to define a consensus PD virome.

#### 3.3.3 Potential biological relevance

The main mechanistic importance of the virome likely lies in phage–bacteria interactions. PD-associated vOTUs have been predicted to infect a broad range of bacterial genera, including *Alistipes*, *Oscillibacter*, *Faecalibacterium*, *Agathobaculum*, and *Lawsonibacter*, as well as members of the Oscillospiraceae family, whereas control-enriched vOTUs are more commonly associated with *Bacteroides* and *Prevotella* (Chen et al., 2022b). Notably, several of these taxa, including *Faecalibacterium*, *Agathobaculum*, *Bacteroides*, and *Prevotella*, are key SCFA producers, raising the possibility that phage-mediated ecological pressure influences microbial turnover, metabolic output, and gut barrier integrity (Chen et al., 2022b; Mirzaei et al., 2021; Hester et al., 2015; Manghi et al., 2024). More broadly, phages can alter bacterial competition, favor lytic or lysogenic states, and indirectly shape inflammatory tone (Tetz et al., 2018; Tetz and Tetz, 2017, 2016; Karst, 2016). For example, bacteriophage-driven disruption of the microbiota has been shown to increase intestinal permeability *in vivo*, linking phage activity to compromised gut barrier integrity (Tetz and Tetz, 2016). In addition, interactions between commensal bacteria and enteric viruses can enhance viral infectivity and modulate host immune responses (Karst, 2016). Taken together, these observations support the view that virome changes may amplify or stabilize bacterial dysbiosis rather than simply mirror it.

#### 3.3.4 Key knowledge gaps

Despite these signals, the virome literature remains constrained by small sample sizes, incomplete reference databases, inconsistent analytical frameworks, and limited functional validation (Bedarf et al., 2017; Chen et al., 2026; Spencer et al., 2022). At present, virome findings are best interpreted as suggestive evidence that the viral component of the gut ecosystem is altered in PD, but not yet as a mature or independently validated biomarker layer.

### 3.4 Gut mycobiome in PD

#### 3.4.1 Definition and scope

The gut mycobiome refers to the fungal component of the intestinal microbiota. Compared with bacteria and metabolites, the mycobiome remains one of the least studied omics domains in PD, and current evidence is derived from only a small number of human datasets (De Pablo-Fernandez et al., 2022). As a result, fungal findings are inherently more exploratory and should be interpreted with caution.

#### 3.4.2 Observational evidence

The main available study integrating fecal metabolomics with fungal profiling reported that fungal abundance in the gut was low in both PD and control samples relative to bacterial populations. Even so, PD was associated with altered relative representation of several fungal taxa and with increased fungal diversity (De Pablo-Fernandez et al., 2022). Reported shifts included enrichment of genera such as *Hanseniaspora*, *Kazachstania*, and *Penicillium*, together with reduced *Saccharomyces* (De Pablo-Fernandez et al., 2022). Because total fungal burden was low and many detected taxa, including *Saccharomyces*, *Penicillium*, *Agaricus*, *Cladosporium*, *Debaryomyces*, and *Candida*, are commonly associated with dietary or environmental exposure, these findings are better viewed as evidence of altered ecological balance than as proof of a stable PD-specific fungal signature.

#### 3.4.3 Potential biological relevance

Although mechanistic data remain limited, the available study linked fungal alterations to changes in microbial metabolites, including reduced SCFAs and altered volatile organic compounds (De Pablo-Fernandez et al., 2022). These associations raise the possibility that fungi contribute to cross-kingdom metabolic interactions and may influence the inflammatory and chemical environment of the gut. However, the current evidence is observational only and cannot establish whether fungal shifts are primary contributors to PD-related dysbiosis or secondary consequences of broader bacterial and metabolic change.

#### 3.4.4 Key knowledge gaps

The mycobiome literature in PD is currently restricted by very small study numbers, limited replication, and uncertainty regarding whether detected fungi represent persistent colonizers or transient exposures (De Pablo-Fernandez et al., 2022). There are essentially no longitudinal, mechanistic, or interventional mycobiome studies in PD, making this a major area for future work.

### 3.5 Gut microbiome-related metabolome in PD

#### 3.5.1 Definition and scope

The gut microbiome-related metabolome refers to the set of small molecules that reflect microbial activity, host metabolism, and host–microbe co-metabolic interactions. These metabolites provide one of the most direct functional links between gut ecology and host physiology. In PD, metabolomic studies have expanded substantially and now complement bacterial profiling by identifying biochemical outputs associated with dysbiosis, inflammation, intestinal permeability, and symptom burden (Tan et al., 2021; Augustin et al., 2023; Hensen and Thiele, 2025). Compared with taxonomy alone, metabolite profiles often offer a more mechanistically interpretable readout of host–microbe interactions.

#### 3.5.2 Observational evidence: SCFAs and broader metabolic reprogramming

The best-established metabolite finding in PD is disruption of SCFA biology. Across multiple cohorts, fecal butyrate and total SCFA concentrations are generally reduced, in parallel with depletion of SCFA-producing bacteria such as *Faecalibacterium*, *Roseburia*, and *Blautia*, and with associations to constipation, inflammation, and disease severity (Aho et al., 2021; Chen et al., 2022a; De Pablo-Fernandez et al., 2022; Augustin et al., 2023). Blood-based SCFA patterns appear more heterogeneous, suggesting that altered intestinal production may coexist with changes in absorption, systemic exposure, or host handling (Shin et al., 2020; Wu et al., 2022; Liao et al., 2024). In turn, this heterogeneity likely reflects broader metabolic reprogramming rather than SCFA production alone. Plasma and serum SCFA studies have reported divergent circulating patterns, while other work points to impaired gut barrier integrity and reduced colonic SCFA receptor expression in PD (Shin et al., 2020; Wu et al., 2022; Liao et al., 2024). Beyond SCFAs, metabolomic studies have described alterations in bile acids, indole compounds, urolithins, caffeine-related pathways, aromatic amino acid metabolites, and compounds linked to proteolytic fermentation, again suggesting broad metabolic remodeling rather than a single-pathway abnormality (Tan et al., 2021; Romo-Vaquero et al., 2022; Chen et al., 2025; De Pablo-Fernandez et al., 2022; Augustin et al., 2023; Kumari et al., 2020; Hensen and Thiele, 2025).

#### 3.5.3 Mechanistic pathways

Metabolite findings support several interconnected mechanisms linking gut microbial activity to host physiology in PD. Lower luminal SCFA availability may weaken epithelial integrity and reduce anti-inflammatory signaling, whereas altered circulating metabolite profiles, including changes in circulating SCFAs and markers associated with gut barrier dysfunction and inflammation, may reflect increased gut permeability and systemic immune exposure (Aho et al., 2021; Chen et al., 2022a; Augustin et al., 2023). Additional shifts in bile acid and aromatic amino acid derivatives further suggest a transition from saccharolytic toward more proteolytic and inflammatory microbial metabolism (Cirstea et al., 2020; Tan et al., 2021; Hensen and Thiele, 2025).

Building on these local and systemic disruptions, metabolites may bridge the gut–brain axis through three interconnected routes (Tan et al., 2024). First, at the local intestinal mucosal interface, microbial metabolites act as signaling molecules to modulate enteroendocrine cells and the enteric nervous system, serving as a primary local sensory interface. Second, through immune-mediated pathways, the breakdown of the gut barrier allows pro-inflammatory microbial products to enter the systemic circulation, priming peripheral immune responses that may subsequently propagate neuroinflammatory signaling across the blood–brain barrier (Augustin et al., 2023; Tan et al., 2021). Finally, via systemic humoral transport, circulating metabolites, including altered aromatic amino acid derivatives and bile acids, interface with host metabolic networks, potentially altering oxidative stress–related processes and the availability of key metabolic intermediates linked to neurotransmitter pathways (Hensen and Thiele, 2025; Tan et al., 2024).

Taken together, these pathways may influence enteroendocrine signaling, immune cell metabolism, oxidative stress, and neurotransmitter-related processes, positioning metabolic reprogramming as a central mediator of the functional gut–brain axis in PD (Tan et al., 2024; Augustin et al., 2023; Hensen and Thiele, 2025).

#### 3.5.4 Interventional evidence

Metabolite-directed intervention studies reinforce the idea that the PD gut environment remains at least partially plastic. Resistant starch, prebiotics, probiotics, and direct SCFA-related supplementation have been associated with increased fecal butyrate, reduced fecal inflammatory markers, altered microbial functional pathways, and, in some studies, improvement in non-motor or motor outcomes (Becker et al., 2022; Hall et al., 2023; Petrov et al., 2025; Hegelmaier et al., 2026; Leta et al., 2025). Although these data remain early and heterogeneous, they provide some of the clearest evidence that microbiome-targeted interventions can shift disease-relevant biochemical outputs in humans with PD.

#### 3.5.5 Transitional summary

Taken together, metabolite studies suggest that the PD gut–brain axis is functionally characterized by impaired fermentation capacity, altered host–microbe co-metabolism, and systemic biochemical consequences. Among the omics layers reviewed here, metabolites may provide the most immediate bridge between microbial ecology and clinical phenotype (Tan et al., 2021; Augustin et al., 2023; Hensen and Thiele, 2025). This is because metabolites offer a direct functional readout of microbial activity, capturing active biochemical output rather than taxonomic composition alone. Although microbial community structure may vary across cohorts, recurring metabolite changes, including depletion of SCFAs and shifts toward proteolytic byproducts, provide a more convergent molecular link to intestinal dysfunction and systemic inflammation (Tan et al., 2021; Augustin et al., 2023). Moreover, because metabolites circulate systemically, they integrate host–microbe co-metabolic processes and offer a dynamic snapshot of gut–brain interactions that may be closely tied to disease-related phenotypes (Hensen and Thiele, 2025).

### 3.6 Gut microbiome-related proteome in PD

#### 3.6.1 Definition and scope

Protein-level evidence relevant to the PD gut microbiome is less mature than the corresponding bacterial and metabolite literature and remains relatively sparse. In this review, we focus primarily on microbial or microbially influenced proteins that may participate in gut-origin models of neurodegeneration, while considering host proteomic signals only when they provide indirect support for inflammation- and proteostasis-related pathways linked to the gut microbiome (Hällqvist et al., 2024; Villette et al., 2025; Wojciechowska et al., 2024). This framing distinguishes microbiome-related proteome findings from the broader field of general PD proteomics.

#### 3.6.2 Observational evidence

Direct observational evidence on microbiome-related protein changes in PD is limited, and much of it comes from integrated omics studies rather than dedicated gut proteomic or metaproteomic datasets. Integrated multi-omics analyses have identified microbial functional signatures in prodromal and manifest PD involving glutamate metabolism, flagellar pathways, and immune-relevant transcriptional programs, suggesting that the intestinal ecosystem may become permissive for proteostatic stress before or during early neurodegenerative change (Villette et al., 2025). In parallel, plasma proteomic studies have reported circulating signatures associated with early or prodromal PD, including complement components (e.g., C3, MASP2, SERPING1), coagulation regulators (e.g., SERPINF2), and unfolded protein response markers (e.g., HSPA5) (Hällqvist et al., 2024). Several of these proteins also correlate with motor severity or cognitive decline, indicating that inflammatory and proteostatic stress pathways are active early in disease (Hällqvist et al., 2024). Although these host-derived observations do not establish a gut origin, they provide supportive context for interpreting microbiome-related protein mechanisms.

#### 3.6.3 Potential biological relevance

The strongest microbiome-related protein mechanisms in PD involve the possibility that microbial products help shape a local environment permissive to misfolding, inflammation, and gut–brain signaling. At a broad mechanistic level, gut-origin models of PD propose that misfolded *α*-synuclein may arise or be amplified in the enteric environment and subsequently propagate toward the central nervous system (Braak et al., 2003). Within this framework, bacterial functional amyloids are especially notable. Computational and experimental studies indicate that amyloid-like bacterial proteins, particularly curli fibers composed of the major subunit CsgA, can interact with host amyloidogenic proteins and promote *α*-synuclein aggregation through structural cross-seeding (Wang et al., 2021; Wojciechowska et al., 2024; Burdukiewicz et al., 2023; Golan et al., 2022). In PD-associated gut metagenomes, bacterial functional amyloids, including CsgA homologs, appear enriched relative to controls and have been linked to immune activation pathways, including Toll-like receptor signaling (Wojciechowska et al., 2024). Although much of this evidence remains indirect, it provides one of the clearest microbiome-specific protein mechanisms by which gut microbial activity could intersect with canonical PD pathology.

#### 3.6.4 Key knowledge gaps

Despite growing interest, the microbiome-related proteome field remains limited by the scarcity of direct human gut proteomic and metaproteomic evidence (Hällqvist et al., 2024; Villette et al., 2025; Wojciechowska et al., 2024). Most available studies infer protein-level relevance from integrated multi-omics analyses, plasma proteomics, metatranscriptomics, metagenomic functional prediction, or computational approaches rather than directly quantifying microbial proteins in the gut. In addition, unlike the bacteriome and metabolome literature, this field currently offers little direct interventional evidence in humans with PD. As a result, microbiome-related protein findings remain more useful for hypothesis generation and mechanistic framing than for firm causal inference, and the microbial protein dimension of PD remains substantially less developed than the bacteriome and metabolome literature.

### 3.7 Integrated synthesis across omics layers

To facilitate direct comparison across omics domains, **Table 1** summarizes the relative depth of evidence, recurrent findings, cross-omics concordance, and major limitations identified across Sections 3.2–3.6. The clearest cross-omics convergence is observed between the bacteriome and metabolome, particularly the depletion of SCFA-producing taxa alongside reduced fecal SCFA levels and broader metabolic remodeling. In contrast, evidence from the virome, mycobiome, and proteome remains comparatively limited and exploratory, and several findings, including the direction of viral family-level changes, individual fungal taxa, and circulating SCFA patterns, remain heterogeneous across studies.

**Table 1:** Comparative synthesis of recurrent findings and cross-omics concordance across omics domains in Parkinson’s disease.

Omics domain	Evidence base	Recurrent findings	Cross-omics concordance	Main limitations
Bacteriome	39 entries	Depletion of SCFA-producing taxa (*Faecalibacterium*, *Roseburia*, *Blautia*); enrichment of *Akkermansia*; altered microbial fermentation and functional capacity.	Consistent with reduced fecal SCFAs and findings related to barrier dysfunction and inflammatory signaling.	Individual taxa and effect sizes vary across cohorts; substantial geographic, dietary, medication-related, and analytical heterogeneity.
Virome	3 entries	Altered viral community structure, differential vOTUs, and changes in bacteriophage richness and composition.	PD-associated phages are linked to bacterial hosts including SCFA-producing taxa, suggesting connections between phage ecology, bacterial structure, and metabolic output.	Few cohorts, small sample sizes, incomplete reference databases, heterogeneous pipelines, and limited functional validation.
Mycobiome	3 entries	Altered fungal composition and diversity, with changes in several fungal genera.	Fungal changes co-occur with reduced SCFAs and altered volatile organic compounds, suggesting ecological changes accompanying metabolic dysregulation.	Very limited replication, low fungal biomass, and uncertainty regarding stable colonization versus transient exposure.
Metabolome	20 entries	Reduced fecal butyrate and total SCFAs; altered bile acid, amino acid, and proteolytic metabolism.	Consistent with depletion of SCFA-producing bacteria and provides a functional link between microbial dysbiosis, barrier integrity, and immune signaling.	Circulating SCFA findings are heterogeneous; results vary by biospecimen, diet, medication exposure, and analytical platform.
Proteome	3 entries	Altered complement, inflammatory, and unfolded-protein-response pathways; potential microbial amyloid-related mechanisms.	Compatible with systemic immune and proteostatic stress; bacterial functional amyloids may link microbial signals to *α*-synuclein aggregation.	Sparse direct human gut proteomic evidence; substantial reliance on systemic proteomics and mechanistic inference.

Across omics domains, the most consistent pattern is not a single microbial signature but a convergent model of gut ecosystem disruption in PD. Bacterial studies indicate depletion of fermentative commensals and enrichment of mucin-degrading or inflammation-associated taxa; virome studies suggest altered phage ecology; mycobiome data point to cross-kingdom ecological disturbance; metabolomic studies show reduced SCFA output and broader host–microbe metabolic remodeling; and protein-focused work raises the possibility of immune activation and amyloid-related mechanisms linked to the intestinal environment (Nishiwaki et al., 2020; Romano et al., 2021; Tan et al., 2021; De Pablo-Fernandez et al., 2022; Augustin et al., 2023; Hällqvist et al., 2024; Villette et al., 2025; Wojciechowska et al., 2024). Together, these findings support a multi-layer view of the PD gut–brain axis in which altered microbial ecology, metabolic dysfunction, barrier impairment, immune activation, and proteostatic stress may be interdependent rather than separate processes.

A clear example of cross-omics convergence is the short-chain fatty acid (SCFA) axis. Across bacteriome studies, depletion of SCFA-producing taxa such as *Faecalibacterium*, *Roseburia*, and *Blautia* repeatedly parallels metabolomic evidence of reduced fecal butyrate and total SCFA concentrations. Aho et al. (2021) further examined microbial composition, fecal SCFAs, inflammatory markers, and intestinal permeability markers within the same participants, placing these features within a shared disturbed intestinal environment (Aho et al., 2021). Integrated metagenomic and metabolomic evidence further supports convergence between changes in microbial composition and function and altered metabolic output (Nishiwaki et al., 2024). Virome findings add another potential layer to this pattern, as PD-associated viruses have been linked to bacterial hosts that include SCFA-producing taxa, suggesting that altered phage ecology may influence the same bacterial–metabolic network (Chen et al., 2026). Similarly, combined mycobiome and metabolomic profiling identified fungal compositional changes that co-occurred with reduced SCFAs and altered volatile organic compounds (De Pablo-Fernandez et al., 2022). Together, these observations identify the SCFA-related ecological–metabolic axis as one of the clearest areas of cross-omics convergence in the current literature.

By contrast, several frequently reported findings remain less clearly connected across omics layers. *Akkermansia* enrichment is recurrent in bacteriome studies, but direct within-cohort multi-omics evidence linking this taxonomic shift to proposed consequences such as mucus degradation or barrier dysfunction remains limited. Likewise, host proteomic signatures involving complement, inflammatory, and unfolded-protein-response pathways have not yet been directly linked to specific gut microbial drivers (Hällqvist et al., 2024). Evidence for bacterial amyloid-mediated *α*-synuclein cross-seeding provides a plausible protein-level mechanism, but is derived primarily from experimental models rather than direct human gut metaproteomic observations. These gaps illustrate where integrated multi-omics studies are particularly needed to distinguish correlated disease signatures from mechanistically connected pathways.

## 4 Discussion

This review highlights how the microbiome literature in Parkinson’s disease (PD) has moved beyond a narrow focus on bacterial composition and now supports a broader, multi-layer model of gut ecosystem disruption. Across bacteria, viruses, fungi, metabolites, and proteins, the most consistent signal is not a single disease-defining organism or pathway, but a convergent pattern linking altered microbial ecology with impaired fermentation, barrier dysfunction, immune activation, and protein-centered mechanisms relevant to neurodegeneration. Even so, the strength of evidence remains uneven. Bacterial microbiome and metabolome studies currently provide the most robust and replicated findings, whereas virome, mycobiome, and proteome observations remain earlier and more hypothesis-generating.

### 4.1 Principal findings

Several principal findings emerge from the current evidence base. First, although the specific bacterial taxa and effect sizes vary across cohorts, several compositional patterns recur across PD microbiome studies. In particular, enrichment of *Akkermansia* and depletion of SCFA-producing taxa, including *Faecalibacterium*, *Roseburia*, and *Blautia*, have been repeatedly reported across meta-analyses and multicohort studies (Nishiwaki et al., 2020; Romano et al., 2021; Boktor et al., 2023). Second, metabolomic studies indicate that these ecological shifts are functionally meaningful, particularly through reduced SCFA production and broader changes in host–microbe co-metabolism (Tan et al., 2021; Augustin et al., 2023; Hensen and Thiele, 2025). Third, emerging virome and mycobiome studies suggest that PD-associated gut alterations extend beyond the bacteriome. Virome studies have reported changes in viral community structure and phage–bacteria associations involving bacterial hosts that include SCFA-producing taxa, whereas mycobiome studies have identified altered fungal composition co-occurring with reduced SCFAs and changes in volatile organic compounds (Bedarf et al., 2017; Chen et al., 2026; De Pablo-Fernandez et al., 2022). These findings suggest potential interactions among microbial and metabolic components of the intestinal ecosystem; however, the available evidence remains limited and largely observational and does not establish direct causal relationships. Finally, protein-level findings, including host proteomic signatures and bacterial functional amyloids, extend the field toward mechanisms that could connect gut dysfunction with inflammation, proteostatic stress, and *α*-synuclein pathology (Hällqvist et al., 2024; Villette et al., 2025; Wojciechowska et al., 2024). These findings should also be considered in light of the clinical and genetic heterogeneity of PD. Most microbiome studies reviewed here primarily enrolled patients with idiopathic or sporadic PD and did not systematically stratify participants according to monogenic or familial forms of disease. Accordingly, the microbiome alterations summarized in this review are most directly relevant to sporadic PD, in which environmental exposures, diet, gastrointestinal physiology, medication use, and host susceptibility may interact with the gut ecosystem. In contrast, evidence specifically addressing hereditary or monogenic PD remains limited, and it is not yet possible to determine whether these forms are associated with distinct or comparable microbiome alterations. Genetic susceptibility and microbiome-related mechanisms are also not necessarily mutually exclusive, as PD-associated genetic variants may influence intestinal immune function or host–microbe interactions. Future studies directly comparing sporadic PD with genetically defined PD subgroups will therefore be needed to determine whether microbiome signatures are shared across disease etiologies or are predominantly associated with sporadic disease.

### 4.2 From taxa to function

One of the clearest conceptual advances in the field is the shift from descriptive dysbiosis toward functional interpretation. Early microbiome studies in PD were framed largely in terms of taxonomic differences, but more recent work suggests that functional output may be more informative than composition alone (Tan et al., 2021; Villette et al., 2025). Reduced abundance of butyrate-producing bacteria, lower fecal SCFA concentrations, altered bile acid and amino acid metabolism, and evidence of increased proteolytic fermentation together suggest that the PD gut environment is metabolically reprogrammed rather than merely compositionally imbalanced (Cirstea et al., 2020; Aho et al., 2021; Chen et al., 2022a; Augustin et al., 2023). This functional framing matters because it offers a clearer link to biological consequences, including barrier integrity, mucosal immunity, enteroendocrine signaling, and systemic inflammatory tone.

This perspective also helps explain why purely taxonomic biomarkers have been difficult to translate. The same functional state may arise through somewhat different taxonomic configurations depending on geography, diet, medication exposure, and baseline microbial ecology. A function-centered model therefore better accommodates inter-cohort variability while preserving the recurring biological themes that appear most relevant to PD.

### 4.3 Cross-omics integration and the gut–brain axis

Taken together, the reviewed evidence supports a cross-omics model of the PD gut–brain axis in which microbial ecology, metabolic function, barrier integrity, immune signaling, and proteostasis are interconnected rather than independent processes. Bacterial dysbiosis provides the most established ecological component, particularly the depletion of SCFA-producing taxa such as *Faecalibacterium*, *Roseburia*, and *Blautia*, together with enrichment of mucin-degrading organisms such as *Akkermansia* (Nishiwaki et al., 2020; Romano et al., 2021; Boktor et al., 2023). These compositional changes converge with metabolomic findings showing reduced fecal SCFAs and broader alterations in host– microbe co-metabolism. Microbiota composition, fecal SCFAs, inflammatory markers, and intestinal permeability markers have been examined within the same participants (Aho et al., 2021), while shotgun metagenomic analysis combined with fecal SCFA and polyamine profiling has identified coordinated taxonomic, functional, and metabolic abnormalities (Nishiwaki et al., 2024).

The virome and mycobiome may modify this ecological–metabolic axis rather than represent entirely separate pathways. PD-associated bacteriophages have been linked to bacterial hosts that include SCFA-producing taxa, suggesting that phage-mediated ecological pressure could influence microbial turnover and metabolic output (Chen et al., 2026). Experimental studies provide a causal precedent for such phage–bacteria interactions: bacteriophage-driven disruption of bacterial communities has been shown *in vivo* to increase intestinal permeability, demonstrating that phage activity can alter bacterial ecology and compromise gut barrier integrity (Tetz and Tetz, 2016). Importantly, this causal relationship has not been established specifically in human PD. Similarly, combined mycobiome and metabolomic profiling identified fungal alterations that co-occurred with reduced SCFAs and changes in volatile organic compounds (De Pablo-Fernandez et al., 2022). In contrast to the experimental evidence for phage–bacteria interactions, current PD mycobiome evidence remains observational and does not establish whether fungal changes are causes or consequences of broader bacterial and metabolic dysregulation. Integrated multi-omics profiling has also identified coordinated abnormalities across multiple biological layers in prodromal and idiopathic PD, demonstrating that these alterations can co-occur within the same disease context, although their temporal and causal ordering remains unresolved (Villette et al., 2025).

A major point of convergence across these layers is the intestinal barrier and mucosal immune interface. Reduced SCFA availability and increased mucin degradation may weaken epithelial homeostasis and increase exposure to microbial products, thereby promoting local and systemic inflammatory signaling. Consistent with this framework, increased intestinal permeability in early PD has been associated with bacterial exposure, oxidative stress, and intestinal *α*-synuclein accumulation (Forsyth et al., 2011). Host proteomic findings involving complement, inflammatory, and unfolded-protein-response pathways provide additional evidence of systemic immune and proteostatic stress, although they do not establish a gut origin (Hällqvist et al., 2024). Experimental studies of bacterial functional amyloids provide another mechanistic example: curli/CsgA can promote *α*-synuclein aggregation through cross-seeding, linking a defined microbial product to a protein-level process relevant to PD pathology (Wang et al., 2021). However, the extent to which this mechanism contributes to human PD remains uncertain.

Collectively, these findings support a framework in which changes across microbial, metabolic, barrier, immune, and protein-related processes may interact within the PD gut–brain axis. However, this framework should not be interpreted as a proven linear causal cascade. Direct cause-and-effect evidence for cross-kingdom interactions in human PD remains scarce; most human findings are observational, whereas the clearest causal examples currently come from experimental systems. Longitudinal and integrated multi-omics studies will therefore be required to establish the temporal direction and causal relationships among these processes.

### 4.4 Clinical relevance

From a translational standpoint, the most immediate promise of this literature lies in biomarker development and patient stratification. Multi-omics studies suggest that combining microbial, metabolic, and host-response features may outperform single-layer approaches for distinguishing PD from controls and for identifying clinically meaningful subgroups (Tan et al., 2021; Lin et al., 2025; Lee et al., 2025; Hällqvist et al., 2024). Metabolites may be especially useful in this regard because they provide a functional readout that is both biologically interpretable and, in some cases, measurable in accessible biospecimens.

Interventional studies also support the idea that at least some components of the PD gut ecosystem are modifiable. Probiotics, prebiotics, resistant starch, and SCFA-related interventions have shown short-term effects on constipation, inflammatory markers, microbial function, and selected motor and non-motor outcomes (Becker et al., 2022; Hall et al., 2023; Petrov et al., 2025; Hegelmaier et al., 2026; Leta et al., 2025; Du et al., 2025). However, current data are insufficient to conclude that these interventions modify disease progression. At present, the strongest translational interpretation is that microbiome-targeted strategies may influence disease-relevant physiology and symptoms, but they remain far from validated disease-modifying therapies.

### 4.5 Why the literature remains inconclusive

Despite substantial progress, several limitations continue to constrain interpretation. Most studies are cross-sectional, making it difficult to determine whether observed microbiome alterations are causes, consequences, or correlates of PD. Medication exposure, constipation severity, stool consistency, dietary patterns, and geography remain major confounders and are not uniformly controlled across cohorts (Nishiwaki et al., 2020; Romano et al., 2021; Boktor et al., 2023).

Sample processing, sequencing platforms, and bioinformatic pipelines also vary substantially, which further complicates reproducibility and meta-synthesis.

These limitations are especially pronounced in the virome, mycobiome, and proteome literature. Viral studies remain heavily constrained by incomplete reference databases and methodological inconsistency; fungal studies are few and often difficult to interpret because of low biomass and uncertainty about colonization versus transient exposure; and microbial protein-level mechanisms are often inferred rather than directly measured in human gut samples (Bedarf et al., 2017; De Pablo-Fernandez et al., 2022; Wojciechowska et al., 2024). As a result, the field currently offers stronger support for a general model of gut ecosystem disruption than for any single definitive microbial signature or mechanistic pathway.

### 4.6 Future directions

Future work should prioritize longitudinal, deeply phenotyped, multi-omics cohorts that include prodromal and early-stage PD. Studies that measure bacteria, viruses, fungi, metabolites, and host or microbial proteins within the same individuals will be especially valuable for clarifying temporal relationships and identifying cross-omic interactions (Tan et al., 2021; Villette et al., 2025). Standardization of sample collection, sequencing, analytical pipelines, and confounder adjustment will also be essential if the field is to move toward reproducible biomarkers and comparable intervention studies.

Mechanistic validation should be a second major priority. In particular, the field needs experimental work that links observed human microbial and metabolic signatures to defined effects on barrier function, immune signaling, enteroendocrine pathways, and *α*-synuclein biology. Finally, intervention studies should move beyond small proof-of-concept designs toward biomarker-informed trials that can test whether microbiome modulation produces sustained, mechanistically interpretable changes in disease-relevant pathways.

In summary, current evidence supports the view that PD is associated with disruption of the gut ecosystem across multiple biological layers. The most mature data implicate bacterial dysbiosis and metabolic dysfunction, while newer findings in the virome, mycobiome, and proteome broaden the mechanistic landscape and reinforce the need for an integrated, systems-level approach. The central challenge now is not simply to catalogue more differences between patients and controls, but to determine which microbiome-associated alterations are biologically consequential, clinically useful, and potentially modifiable in the course of PD.

## 5 Conclusions

Current evidence supports the view that Parkinson’s disease is associated with disruption of the gut ecosystem across multiple biological layers, including bacteria, viruses, fungi, metabolites, and proteins. Among these domains, the strongest and most consistent findings implicate bacterial dysbiosis and metabolic dysfunction, particularly reduced short-chain fatty acid production, altered host–microbe co-metabolism, and signatures consistent with barrier impairment and immune activation. Emerging data on the virome, mycobiome, and protein-level mediators broaden this framework and suggest that PD-related gut alterations are best understood as a cross-kingdom and multi-omics phenomenon rather than a bacteria-only process.

At the same time, the field remains limited by cross-sectional study designs, methodological heterogeneity, and uneven evidence depth across omics layers. The major challenge moving forward is not simply to catalogue additional differences between patients and controls, but to identify which microbiome-associated alterations are mechanistically important, clinically informative, and potentially modifiable. Longitudinal, standardized, and integrated multi-omics studies will be essential for determining whether the gut microbiome can contribute meaningfully to biomarker development, patient stratification, and future therapeutic strategies in PD.

## Data Availability

The original contributions presented in the study are included in the article/**Supplementary Material**; the full list of included studies is provided in **Supplementary Table 2**. Code and publication-ready files for the figures are available in the public GitHub repository at https://github.com/hanhuiyeye/PD. An R Shiny application that generates PRISMA 2020 flow diagrams, developed alongside this review, is deployed at https://eok7.shinyapps.io/PRISMA/ (source code: https://github.com/euniceokk/prisma). Further inquiries can be directed to the corresponding author.
