# Supplementary material for: Beyond bacteria: a multi-omics view of the gut–brain axis in Parkinson’s disease
Source: Front Cell Infect Microbiol. 2026 Sep 11;16:1900578. doi: 10.3389/fcimb.2026.1900578 (PMC13612191; doi:10.3389/fcimb.2026.1900578)
Supplement: Supplementary file 1 [file DataSheet1.pdf]

Supplementary Material

1 SUPPLEMENTARY TABLES AND FIGURES

1.1 Supplementary Figure

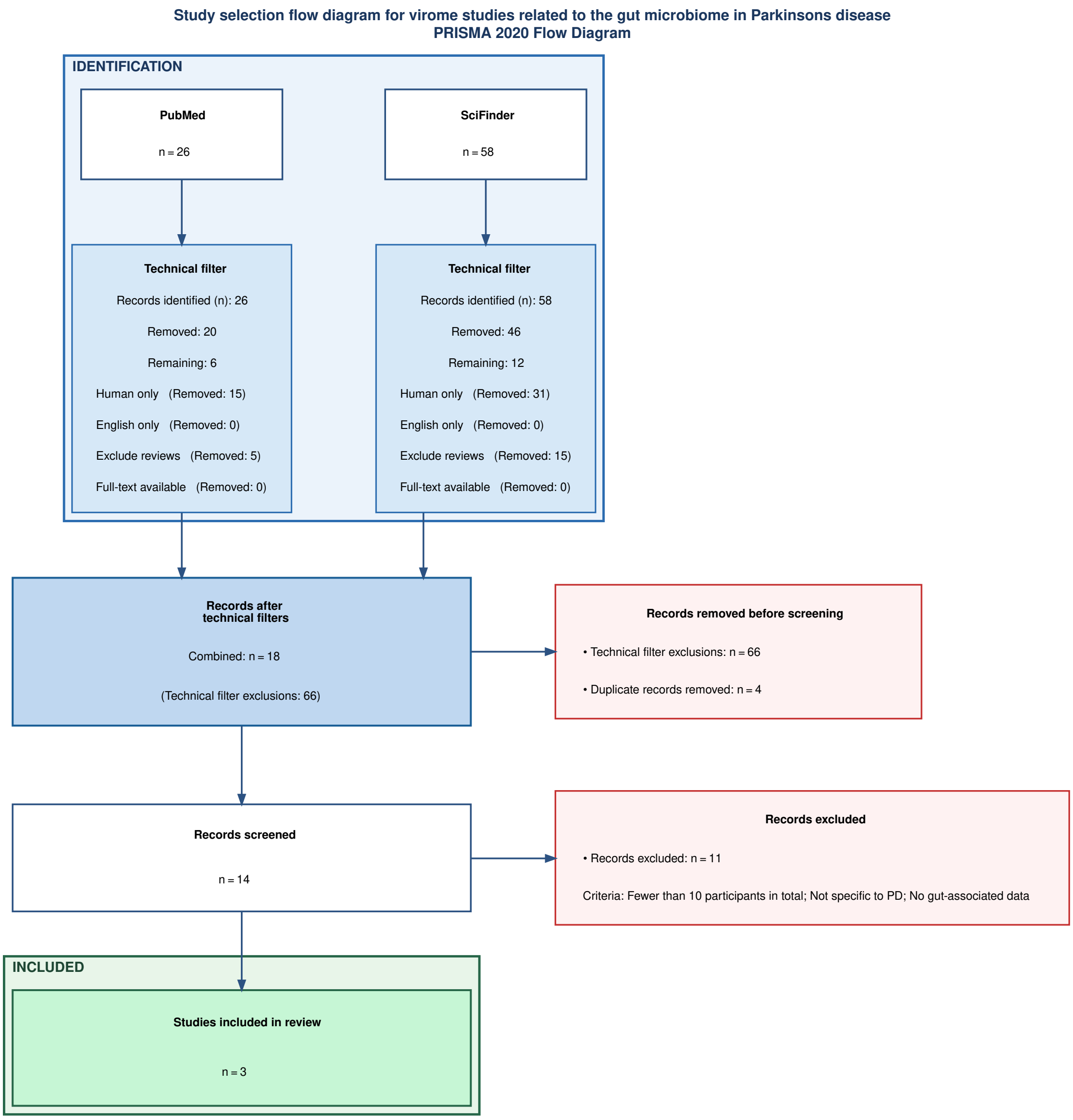

Figure S1. Study selection flow diagram for virome studies related to the gut microbiome in Parkinson's disease. The PRISMA flow diagram summarizes the identification, technical filtering, duplicate removal, and eligibility screening process for gut microbiome-related virome studies in Parkinson's disease. After applying database-specific technical filters and study-level eligibility criteria, 3 studies were included in the review.

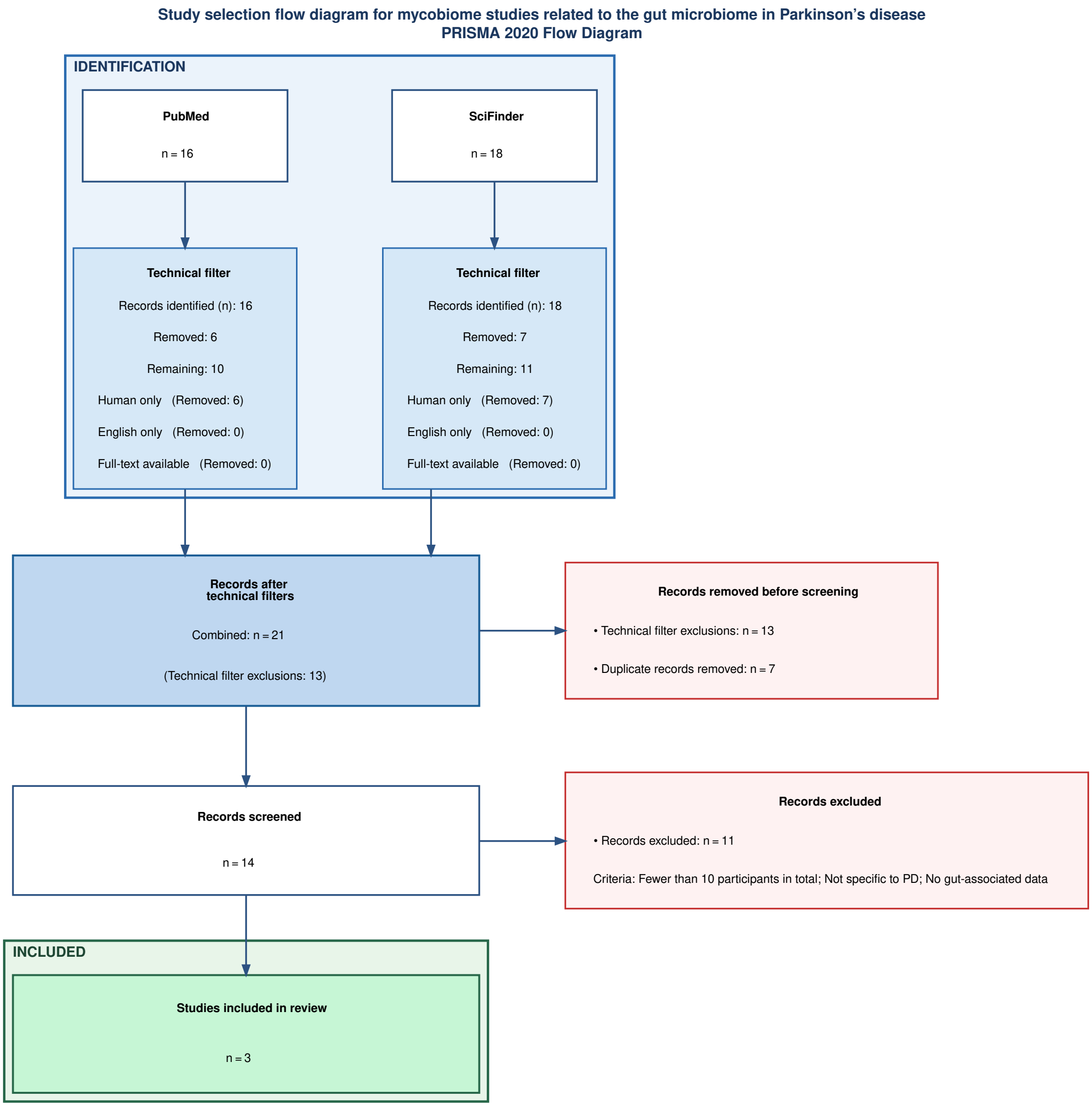

Figure S2. Study selection flow diagram for mycobiome studies related to the gut microbiome in Parkinson's disease. The PRISMA flow diagram summarizes the identification, technical filtering, duplicate removal, and eligibility screening process for gut microbiome-related mycobiome studies in Parkinson's disease. After applying database-specific technical filters and study-level eligibility criteria, 4 studies were included in the review.

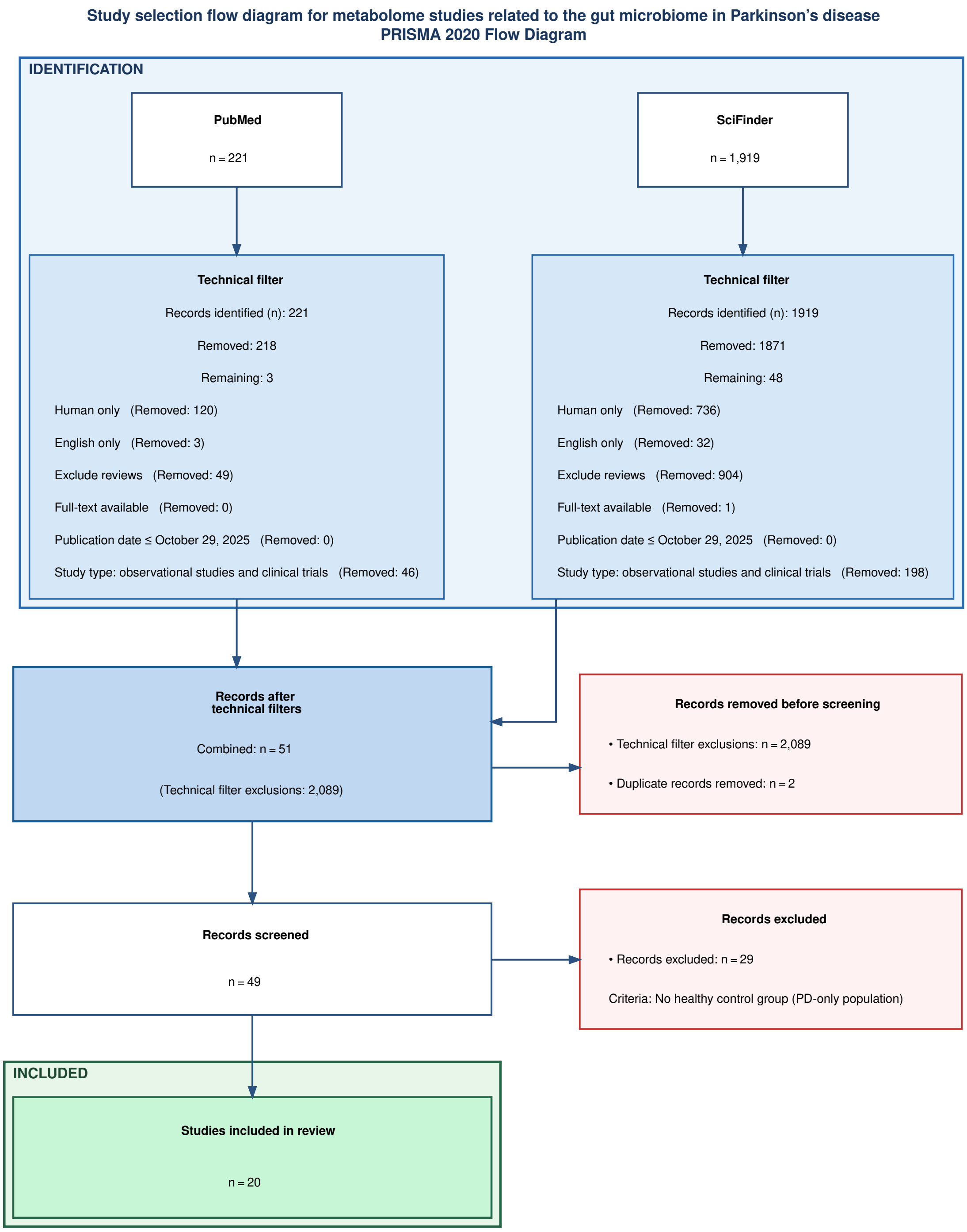

Figure S3. Study selection flow diagram for metabolome studies related to the gut microbiome in Parkinson's disease. The PRISMA flow diagram summarizes the identification, technical filtering, duplicate removal, and eligibility screening process for gut microbiome-related metabolome studies in Parkinson's disease. After applying database-specific technical filters and study-level eligibility criteria, 20 studies were included in the review (15 observational and 5 interventional).

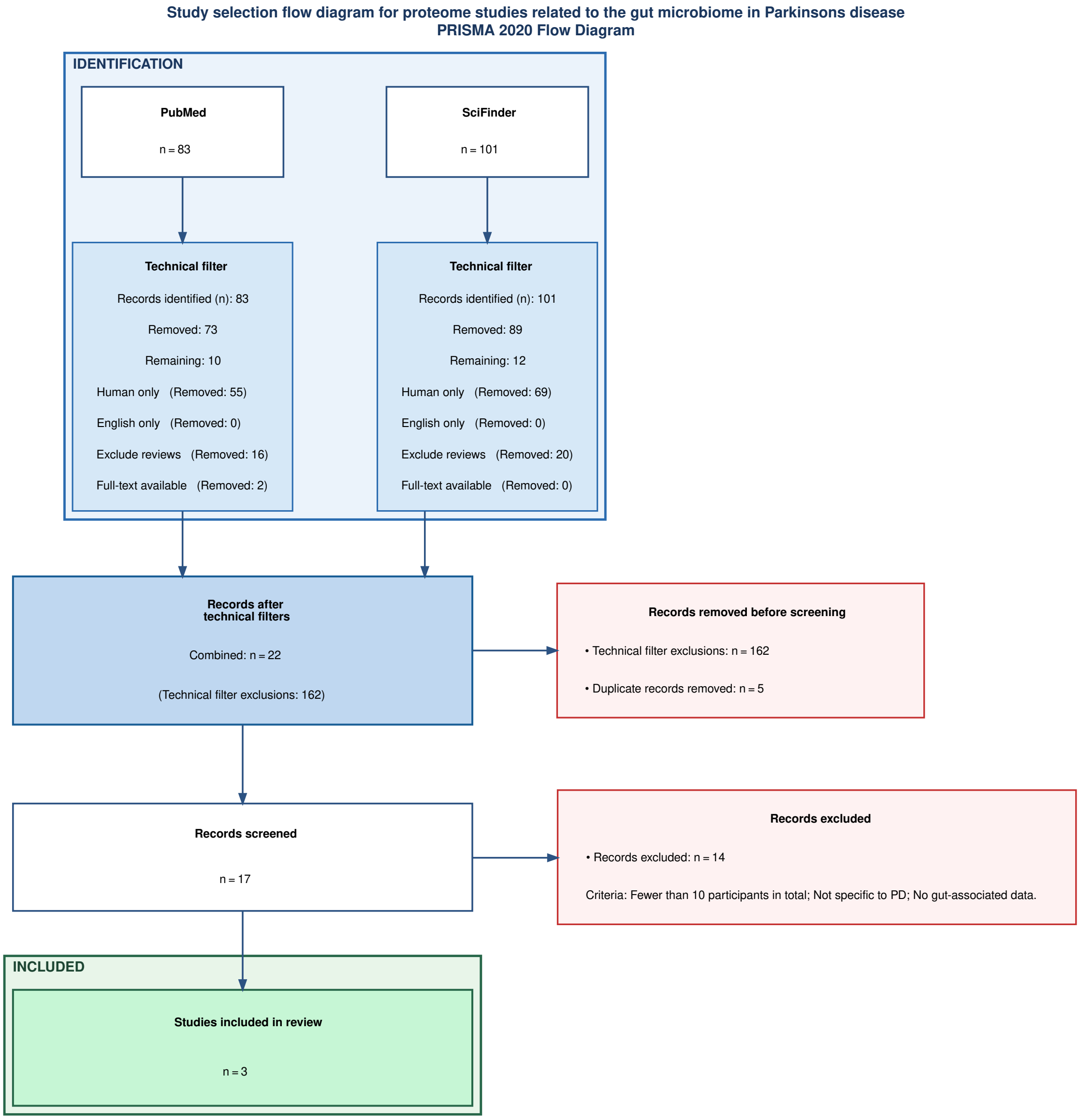

Figure S4. Study selection flow diagram for proteome studies related to the gut microbiome in Parkinson's disease. The PRISMA flow diagram summarizes the identification, technical filtering, duplicate removal, and eligibility screening process for gut microbiome-related proteome studies in Parkinson's disease. After applying database-specific technical filters and study-level eligibility criteria, 3 studies were included in the review.

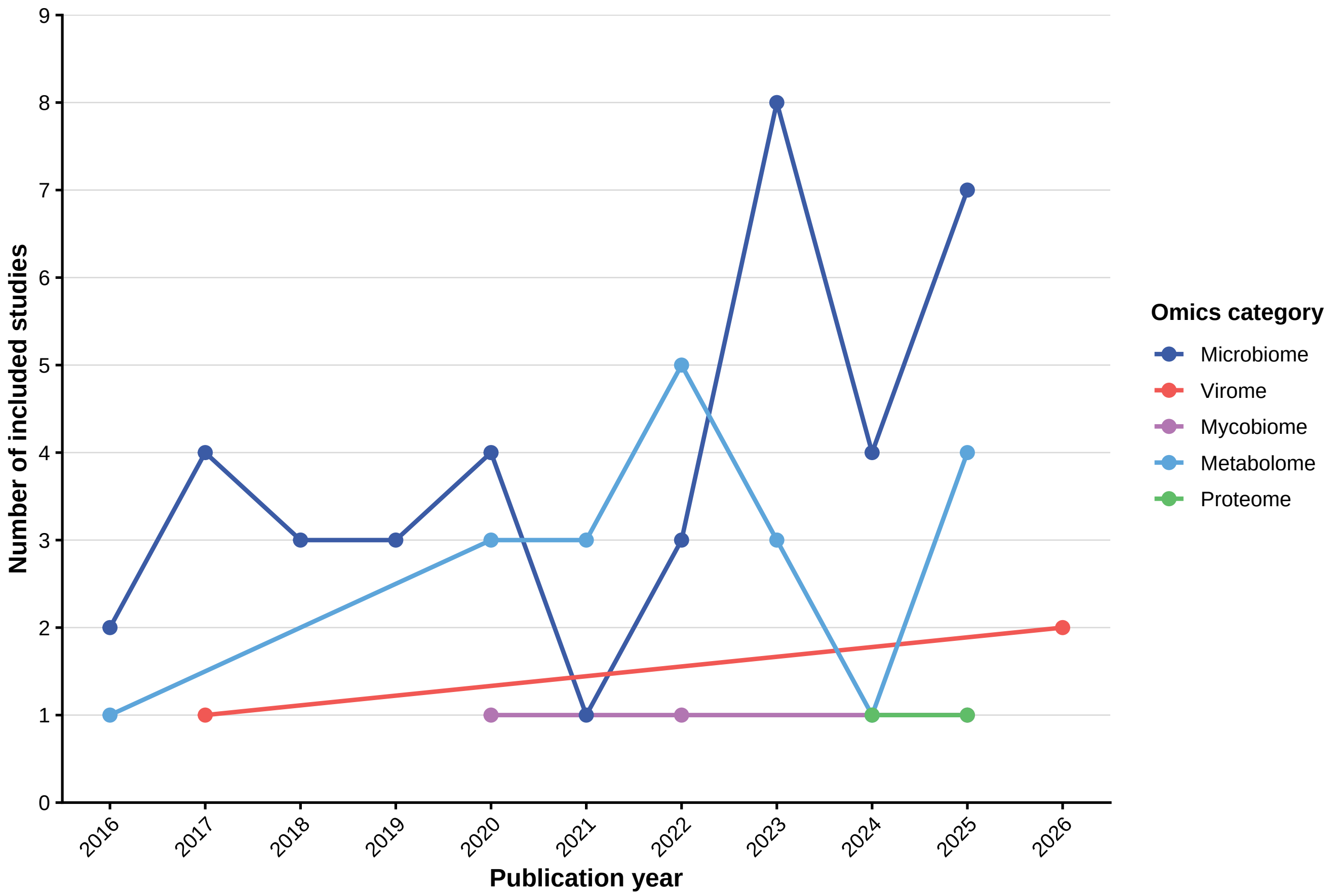

Figure S5. Publication trends of included studies by omics category. Annual number of included studies across microbiome (bacteriome), virome, mycobiome, metabolome, and proteome domains. The figure illustrates the sustained dominance of bacterial microbiome studies, the growth of metabolomic studies in recent years, and the comparatively limited but emerging literature on virome, mycobiome, and proteome-related research in Parkinson's disease.

1.2 Supplementary Tables

1.2.1 Complete Search Strategies

Table S1: Complete free-text retrieval formulas used for each omics domain.

Domain	Complete retrieval formula
**Bacteriome**	`("Parkinson Disease" OR "Idiopathic Parkinson’s Disease" OR "Lewy Body Parkinson’s Disease" OR "Parkinson’s Disease, Idiopathic" OR "Parkinson’s Disease, Lewy Body" OR "Parkinson Disease, Idiopathic" OR "Parkinson’s Disease" OR "Idiopathic Parkinson Disease" OR "Lewy Body Parkinson Disease" OR "Primary Parkinsonism" OR "Parkinsonism, Primary" OR "Paralysis Agitans")`<br><br>`AND`<br><br>`("Gastrointestinal Microbiomes" OR "Microbiome, Gastrointestinal" OR "Gut Microbiome" OR "Gut Microbiomes" OR "Microbiome, Gut" OR "Gut Microbiota" OR "Gut Microbiotas" OR "Microbiota, Gut" OR "Gastrointestinal Microbiota" OR "Gastrointestinal Microbiotas" OR "Microbiota, Gastrointestinal" OR "Gastrointestinal Microbial Community" OR "Gastrointestinal Microbial Communities" OR "Microbial Community, Gastrointestinal" OR "Gastric Microbiome" OR "Gastric Microbiomes" OR "Microbiome, Gastric" OR "Intestinal Microbiome" OR "Intestinal Microbiomes" OR "Microbiome, Intestinal" OR "Intestinal Microbiota" OR "Intestinal Microbiotas" OR "Microbiota, Intestinal")`
**Mycobiome**	`("Parkinson Disease" OR "Idiopathic Parkinson’s Disease" OR "Lewy Body Parkinson’s Disease" OR "Parkinson’s Disease, Idiopathic" OR "Parkinson’s Disease, Lewy Body" OR "Parkinson Disease, Idiopathic" OR "Parkinson’s Disease" OR "Idiopathic Parkinson Disease" OR "Lewy Body Parkinson Disease" OR "Primary Parkinsonism" OR "Parkinsonism, Primary" OR "Paralysis Agitans")`<br><br>`AND`<br><br>`("Mycobiome" OR "Fungal Microbiome" OR "Gut Fungi" OR "Intestinal Fungi" OR "Gut Yeast" OR "ITS Sequencing" OR "Candida" OR "Aspergillus" OR "Saccharomyces")`
**Virome**	`("Parkinson Disease" OR "Idiopathic Parkinson’s Disease" OR "Lewy Body Parkinson’s Disease" OR "Parkinson’s Disease, Idiopathic" OR "Parkinson’s Disease, Lewy Body" OR "Parkinson Disease, Idiopathic" OR "Parkinson’s Disease" OR "Idiopathic Parkinson Disease" OR "Lewy Body Parkinson Disease" OR "Primary Parkinsonism" OR "Parkinsonism, Primary" OR "Paralysis Agitans")`<br><br>`AND`<br><br>`("Virome" OR "Gut Virome" OR "Viral Metagenomics" OR "Enteric Viruses" OR "Bacteriophages" OR "Phages" OR "Gut Viruses")`
**Metabolome**	`("Parkinson Disease" OR "Idiopathic Parkinson’s Disease" OR "Lewy Body Parkinson’s Disease" OR "Parkinson’s Disease, Idiopathic" OR "Parkinson’s Disease, Lewy Body" OR "Parkinson Disease, Idiopathic" OR "Parkinson’s Disease" OR "Idiopathic Parkinson Disease" OR "Lewy Body Parkinson Disease" OR "Primary Parkinsonism" OR "Parkinsonism, Primary" OR "Paralysis Agitans")`<br><br>`AND`<br><br>`("Gut metabolites" OR "Stool metabolites" OR "Short-Chain Fatty Acids" OR "Gut metabolomics" OR "Stool metabolomics" OR "Gut metabolome" OR "Stool metabolome")`

Domain	Complete retrieval formula
**Proteome**	("Parkinson Disease" OR "Idiopathic Parkinson's Disease" OR "Lewy Body Parkinson's Disease" OR "Parkinson's Disease, Idiopathic" OR "Parkinson's Disease, Lewy Body" OR "Parkinson Disease, Idiopathic" OR "Parkinson's Disease" OR "Idiopathic Parkinson Disease" OR "Lewy Body Parkinson Disease" OR "Primary Parkinsonism" OR "Parkinsonism, Primary" OR "Paralysis Agitans")<br>AND<br>("Gut Proteins" OR "Stool Proteins" OR "Microbial Proteins" OR "Gut proteomics" OR "Stool proteomics" OR "Microbial proteomics" OR "Gut Proteome" OR "Stool Proteome" OR "Microbial Proteome" OR "Metaproteomics" OR "Bacterial Proteins")

Note: The table reports the complete free-text retrieval formulas used for each omics domain. All searches were subsequently restricted to human studies during technical filtering. Obvious spacing inconsistencies in the working search-term file were standardized for presentation.

1.2.2 Included Studies

Table S2: **Final list of papers included in the systematic review of gut microbiome-related omics in Parkinson's disease.** The table lists all papers finally selected for inclusion in the review, including study title, omics category, Newcastle–Ottawa Scale (NOS) score when applicable, publication year, study region, and cross-listings. Some multi-omics studies report findings in more than one domain and are therefore listed under each relevant omics category; the "Also listed as" column gives the corresponding entry number. The table contains 68 entries representing 61 unique studies.

No.	Title	Omics category	NOS	Year	Study region	Also listed as
1	Microbiota Composition and Metabolism Are Associated With Gut Function in Parkinson's Disease	Microbiome	7	2020	Canada	#55
2	Gut metagenomics-derived genes as potential biomarkers of Parkinson's disease	Microbiome	8	2020	Mainland China	–
3	Gut microbiota in patients with Parkinson's disease in southern China	Microbiome	7	2018	Mainland China	–
4	Differences in the gut microbiome across typical ageing and in Parkinson's disease	Microbiome	8	2023	Mainland China	–
5	Altered gut microbiota and inflammatory cytokine responses in patients with Parkinson's disease	Microbiome	7	2019	Taiwan	–
6	Parkinson's disease and Parkinson's disease medications have distinct signatures of the gut microbiome	Microbiome	8	2017	United States	–
7	More than constipation - bowel symptoms in Parkinson's disease and their connection to gut microbiota	Microbiome	7	2017	United States	–
8	Gut microbiota in Parkinson's disease: Temporal stability and relations to disease progression	Microbiome	8	2019	Finland	–
9	The nasal and gut microbiome in Parkinson's disease and idiopathic rapid eye movement sleep behavior disorder	Microbiome	8	2017	Germany	–
10	Relationships of gut microbiota, short-chain fatty acids, inflammation, and the gut barrier in Parkinson's disease	Microbiome	7	2021	Finland	#48

Continued on next page

No.	Title	Omics category	NOS	Year	Study country/region	Also listed as
11	Study of the gut microbiome in Egyptian patients with Parkinson's Disease	Microbiome	6	2023	Egypt	–
12	Urolithins: potential biomarkers of gut dysbiosis and disease stage in Parkinson's patients	Microbiome	8	2022	Australia	–
13	Relationships Between Rapid Eye Movement Sleep Behavior Disorder and Parkinson's Disease: Indication from Gut Microbiota Alterations	Microbiome	7	2023	Mainland China	–
14	Alterations of fecal microbiota and plasma metabolome in patients with Parkinson's disease with rapid eye movement sleep disorder	Microbiome	7	2024	Mainland China	–
15	Altered gut microbiota in Parkinson's disease patients/healthy spouses and its association with clinical features	Microbiome	7	2020	Mainland China	–
16	Investigating Plasma Metabolomics and Gut Microbiota Changes Associated With Parkinson Disease	Microbiome	–	2024	Mainland China	–
17	Altered gut microbiota in patients with idiopathic Parkinson's disease: an age-sex matched case-control study	Microbiome	7	2023	Turkey	–
18	Association of Fecal and Plasma Levels of Short-Chain Fatty Acids With Gut Microbiota and Clinical Severity in Patients With Parkinson Disease	Microbiome	8	2016	Taiwan	#46
19	Meta-Analysis of Gut Dysbiosis in Parkinson's Disease	Microbiome	–	2020	Japan	–
20	Unraveling gut microbiota in Parkinson's disease and atypical parkinsonism	Microbiome	8	2018	Italy	–
21	Differences in intestinal microbiota in Parkinson's disease and isolated REM sleep behavior disorder	Microbiome	–	2025	Germany	–
22	Associations between gut microbiota characteristics and non-motor symptoms following pharmacological and surgical treatments in Parkinson's disease patients	Microbiome	7	2024	Poland	–

Continued on next page

No.	Title	Omics category	NOS	Year	Study country/region	Also listed as
23	Short chain fatty acids and gut microbiota differ between patients with Parkinson's disease and age-matched controls	Microbiome	7	2016	Germany	#51
24	Dysbiosis of gut microbiota in a selected population of Parkinson's patients	Microbiome	7	2019	Italy	–
25	Alteration of the fecal microbiota in Chinese patients with Parkinson's disease	Microbiome	8	2018	Mainland China	–
26	Alteration of Gut Microbial Metabolites in the Systemic Circulation of Patients with Parkinson's Disease	Microbiome	8	2023	Taiwan	–
27	Metagenome-assembled microbial genomes from Parkinson's disease fecal samples	Microbiome	8	2024	Finland	–
28	Metagenomics of Parkinson's disease implicates the gut microbiome in multiple disease mechanisms	Microbiome	8	2022	United States	–
29	Metabolic modeling links gut microbiota to metabolic markers of Parkinson's disease	Microbiome	7	2025	United States	–
30	Metagenomics of the Gut Microbiome in Parkinson's Disease: Prodromal Changes	Microbiome	8	2023	United States	–
31	Association between the relative abundance of butyrate-producing and mucin-degrading taxa and Parkinson's disease	Microbiome	6	2025	South Africa	–
32	Bacterial Butyrate in Parkinson's Disease Is Linked to Epigenetic Changes and Depressive Symptoms	Microbiome	7	2022	Finland	#56
33	Anxiety-related gut microbiota alterations in Parkinson's disease: distinct associations compared to healthy individuals	Microbiome	8	2025	United States	–
34	Human gut microbiome gene co-expression network reveals a loss in taxonomic and functional diversity in Parkinson's disease	Microbiome	8	2025	Luxembourg	–

Continued on next page

No.	Title	Omics category	NOS	Year	Study country/region	Also listed as
35	Distinct gut microbiome characteristics and dynamics in patients with Parkinson's disease based on the presence of premotor rapid-eye movement sleep behavior disorders	Microbiome	8	2025	South Korea	–
36	Microbial diversity in drug-naïve Parkinson's disease patients	Microbiome	7	2025	Croatia	–
37	A novel pathway of levodopa metabolism by commensal Bifidobacteria	Microbiome	7	2023	Canada	–
38	Functional implications of microbial and viral gut metagenome changes in early stage L-DOPA-naïve Parkinson's disease patients	Microbiome	8	2017	Germany	#40
39	Integrated Multi-Cohort Analysis of the Parkinson's Disease Gut Metagenome	Microbiome	8	2023	United States	–
40	Functional implications of microbial and viral gut metagenome changes in early stage L-DOPA-naïve Parkinson's disease patients	Virome	5	2017	Germany	#38
41	Metagenomics indicates an interplay of the microbiome and functional pathways in Parkinson's disease	Virome	5	2026	United States & Canada	–
42	Alterations of the gut virome in patients with Parkinson's disease	Virome	5	2026	Mainland China	–
43	The Gut Mycobiome in Parkinson's Disease	Mycobiome	7	2021	Canada	–
44	The faecal metabolome and mycobiome in Parkinson's disease	Mycobiome	8	2022	United Kingdom	#57
45	Interplay of constipation, intestinal barrier dysfunction and fungal exposome in aetiopathogenesis of Parkinson's disease: hypothesis with supportive data	Mycobiome	9	2025	United Kingdom	–
46	Association of Fecal and Plasma Levels of Short-Chain Fatty Acids With Gut Microbiota and Clinical Severity in Patients With Parkinson Disease	Metabolome	7	2022	Mainland	#18

Continued on next page

No.	Title	Omics category	NOS	Year	Study country/region	Also listed as
47	Plasma Short-Chain Fatty Acids in Patients With Parkinson's Disease	Metabolome	6	2020	South Korea	–
48	Relationships of gut microbiota, short-chain fatty acids, inflammation, and the gut barrier in Parkinson's disease	Metabolome	6	2021	Finland	#10
49	Parkinson's Disease Is Associated with Impaired Gut-Blood Barrier for Short-Chain Fatty Acids	Metabolome	7	2022	Mainland China	–
50	Serum short-chain fatty acids and its correlation with motor and non-motor symptoms in Parkinson's disease patients	Metabolome	6	2022	Mainland	–
51	Short chain fatty acids and gut microbiota differ between patients with Parkinson's disease and age-matched controls	Metabolome	7	2016	Germany	#23
52	Impaired gut barrier integrity and reduced colonic expression of free fatty acid receptors in patients with Parkinson's disease	Metabolome	7	2024	Taiwan	–
53	Plasma Metabolic Analysis Reveals the Dysregulation of Short-Chain Fatty Acid Metabolism in Parkinson's Disease	Metabolome	6	2023	Mainland China	–
54	Gut Microbial Ecosystem in Parkinson Disease: New Clinicobiological Insights from Multi-Omics	Metabolome	8	2021	Grand Duchy of Luxembourg	–
55	Microbiota Composition and Metabolism Are Associated With Gut Function in Parkinson's Disease	Metabolome	7	2020	Canada	#1
56	Bacterial Butyrate in Parkinson's Disease Is Linked to Epigenetic Changes and Depressive Symptoms	Metabolome	7	2022	Finland	#32
57	The faecal metabolome and mycobiome in Parkinson's disease	Metabolome	7	2022	United Kingdom	#44
58	Faecal metabolite deficit, gut inflammation and diet in Parkinson's disease: Integrative analysis indicates inflammatory response syndrome	Metabolome	8	2023	United Kingdom	–

Continued on next page

No.	Title	Omics category	NOS	Year	Study country/region	Also listed as
59	Quantitative metabolomics of saliva using proton NMR spectroscopy in patients with Parkinson's disease and healthy controls	Metabolome	6	2020	India	–
60	Intestinal biomarkers, microbiota composition, and genetic predisposition to inflammatory bowel disease as predictors of Parkinson's disease manifestation	Metabolome	7	2025	Germany	–
61	Resistant starch improves Parkinson's disease symptoms through restructuring of the gut microbiome and modulating inflammation	Metabolome	–	2025	Germany	–
62	Supplementation with short-chain fatty acids and a prebiotic improves clinical outcome in Parkinson's disease: a randomized double-blind prospective study	Metabolome	–	2025	Germany	–
63	Effects of a Four-Strain Probiotic on Gut Microbiota, Inflammation, and Symptoms in Parkinson's Disease: A Randomized Clinical Trial	Metabolome	–	2025	United Kingdom and Sweden	–
64	An open label, non-randomized study assessing a prebiotic fiber intervention in a small cohort of Parkinson's disease participants	Metabolome	–	2023	United States	–
65	Effects of Resistant Starch on Symptoms, Fecal Markers, and Gut Microbiota in Parkinson's Disease - The RESISTA-PD Trial	Metabolome	–	2021	Germany	–
66	Plasma proteomics identify biomarkers predicting Parkinson's disease up to 7 years before symptom onset	Proteome	7	2024	Germany	–
67	Integrated multi-omics highlights alterations of gut microbiome functions in prodromal and idiopathic Parkinson's disease	Proteome	6	2025	Germany	–
68	Aggregating gut: on the link between neurodegeneration and bacterial functional amyloids	Proteome	–	pre-print	Germany & United States	–

Note: NOS scores were assessed only for eligible observational studies. Entries marked "–" indicate studies for which the NOS assessment was not applicable or not available, including randomized or interventional studies, preprints, meta-analyses, or records without an available NOS score.
